# Hot Deformation Behavior and Pulse Current Auxiliary Isothermal Forging of Hot Pressing Sintering TiAl Based Alloys

**DOI:** 10.3390/ma10121437

**Published:** 2017-12-16

**Authors:** Chengcheng Shi, Shaosong Jiang, Kaifeng Zhang

**Affiliations:** 1 School of Materials Science and Engineering, Harbin Institute of Technology, Harbin 150001, China; shicchit@163.com; 2 National Key Laboratory for Precision Hot Processing of Metals, Harbin Institute of Technology, Harbin 150001, China

**Keywords:** TiAl based alloys, hot pressing sintering, hot deformation behavior, microstructure evolution, pulse current auxiliary isothermal forging

## Abstract

This paper focuses on the fabrication of as-forged Ti46.5Al2Cr1.8Nb-(W, B) alloy via pulse current auxiliary isothermal forging (PCIF). The starting material composed of near gamma (NG) microstructure was fabricated by adopting pre-alloyed powders via hot pressing sintering (HPS) at 1300 °C. Isothermal compression tests were conducted at a strain rate range of 0.001–0.1 s^−1^ and a temperature range of 1125–1275 °C to establish the constitutive model and processing map. The optimal hot deformation parameters were successfully determined (in a strain rate range of 10^−3^–2.5 × 10^−3^ s^−1^ and temperature range of 1130–1180 °C) based on the hot processing map and microstructure observation. Accordingly, an as-forged TiAl based alloy without cracks was successfully fabricated by PCIF processing at 1175 °C with a nominal strain rate of 10^−3^ s^−1^. Microstructure observation indicated that complete dynamic recrystallization (DRX) and phase transformation of γ→α_2_ occurred during the PCIF process. The elongation of as-forged alloy was 136%, possessing a good secondary hot workability, while the sintered alloy was only 66% when tested at 900 °C with a strain rate of 2 × 10^−4^ s^−1^.

## 1. Introduction

The γ-TiAl based alloys are considered as one of the most promising substitutes for nickel-based superalloys in the production of low-pressure turbine blades, turbocharger wheels, exhaust valves, etc., owing to their low density, high specific strength, and excellent creep property [1,2,3,4]. In past research, vacuum melting techniques were the conventional route for fabricating TiAl based alloys, and costly hot isostatic pressing (HIP) was essential to eliminate the microscopic cracks, microstructural and chemical composition inhomogeneities caused by the melting processing [5,6,7,8,9]. Casting microstructures of the TiAl based alloys are predominantly composed of α_2_/γ lamellar colonies. Considerable investigations have been carried out to obtain near gamma (NG) TiAl based alloys through forging [8,9,10], hot-pack rolling [11], or the extruding [12] process by adopting casting TiAl based alloys, due to the comparatively good hot deformability of the NG microstructure. However, remnant α_2_/γ lamellar structures, which would compromise the ductility of the TiAl based alloys, are difficult to eliminate completely. Powder metallurgy, such as HIP [13,14] and spark plasma sintering (SPS) [15,16], could produce TiAl based alloys with NG microstructure directly. HIP is a convenient and versatile technique for fabricating TiAl based alloys with NG microstructure by adopting pre-alloyed powders [13,14]. Liu fabricated a Ti45.7Al7Nb0.3W alloy via HIP, and sheets were successfully fabricated by hot rolling based on the investigation of deformation behavior [13,14]. SPS, which densifies powders through the simultaneous application of a pulsed direct current and uniaxial pressure in a few minutes, is an effective way for fabricating TiAl based alloys with fine grains. A. Couret, et al. [15,16] achieved remarkable efforts on the fabrication, microstructure evolution, and mechanical properties of TiAl alloys, accordingly, a near-net shape high-pressure blade was successfully fabricated via SPS technique in recent years. Furthermore, hot pressing sintering (HPS), which has been widely used in fabricating titanium alloy and titanium matrix composites, might be an effective and economical way for fabricating NG TiAl based alloys.

The forging process, which has been widely investigated, is an effective method to improve the performance of TiAl based alloys [8,9,10]. However, TiAl based alloys with narrow hot working windows are characterized by a high proportion of forging defects, such as cracks, adiabatic shear band, localized plastic flow, and coarse grains, etc. Furthermore, past research has shown that deformation parameters have a great influence on the microstructures and mechanical properties. Up to the present, the hot deformation behaviors of the TiAl based alloys except for the NG TiAl based alloys fabricated by the HPS technique, have been widely investigated [17,18,19,20], meanwhile, it is essential to investigate the hot deformation behavior and microstructure evolution of the sintered NG TiAl based alloys in order to obtain as-forged alloy with high-performance. Pulse current auxiliary isothermal forming [21,22,23,24,25,26,27] is a kind of efficient processing method with low energy consumption, and the pulse current can ensure a constant temperature in the forming process to avoid cracks caused by uneven distribution of temperature. However, few works have focused on investigating pulse current auxiliary isothermal forging (PCIF) of TiAl based alloys. 

In this study, TiAl based alloys composed of NG microstructure were fabricated by HPS. The hot deformation characteristics of sintered alloy were investigated, and the constitutive equation and processing map of the sintered alloy was established. Furthermore, a NG TiAl based alloy with uniform dynamic recrystallization microstructure was fabricated through PCIF based on the established processing map and the investigation of microstructure evolution. Tensile tests were carried out to characterize the hot deformability of the forged alloy fabricated by PCIF.

## 2. Materials and Methods 

The experimental material was gas-atomized pre-alloyed powder with nominal composition of Ti-46.5Al-2Cr-1.8Nb-(W, B). The sintered TiAl based alloy bulks were fabricated in a vacuum hot pressing sintering furnace (ZRY55, Jinzhouhangxing, Jinzhou, China) at 1300 °C with a uniaxial pressure of 40 MPa, and a holding time of 120 min, under a vacuum degree of 10^−3^ Pa. 

**Figure 1:**
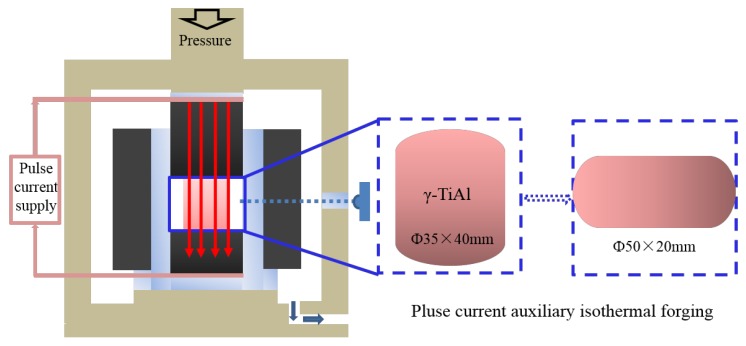
The sketch of pulse current auxiliary isothermal forging (PCIF) equipment (Jinzhouhangxing, China).

Specimens with a diameter of 8 mm and a height of 12 mm were cut through the sintered alloys. The compression tests were performed on a Gleeble-3800D (DSI, Troy, NY, USA) over a temperature range of 1125–1275 °C with an interval of 50 °C and at a strain rate of 0.001, 0.03, 0.01, and 0.1 s^−1^, respectively. For microstructure observation, all specimens were immediately water-quenched after compression. The forged TiAl based alloys were fabricated through PCIF. The starting alloys were sintered at 1300 °C with a diameter of 35 mm and a height of 40 mm. The sintered alloy was forged at 1175 °C with a nominal strain rate of 0.001 s^−1^, and a height reduction of 50%. Figure 1 shows the sketch of PCIF equipment, the pulse power source could provide a 20000 A pulse DC. During the forging process, the diameter of the specimen increased gradually, and the pulse current passing the specimen increased in order to keep a constant temperature. In addition, different nominal strain rates could be obtained by adjusting the hydraulic system.

Tensile tests were carried out on the Instron 5500R electronic universal testing machine (Instron, Norwood, MA, USA) under a strain rate of 2 × 10^−4^ s^−1^. The testing temperatures were 900 °C and 950 °C. Tensile specimens with a gauge section of the 1 mm × 3 mm × 13 mm were machined from the core of the alloys.

Microstructural observation was performed on a Quanta 200FEG scanning electron microscope (SEM, FEI, Hillsboro, OR, USA) equipped with electron backscatter diffraction (EBSD) system. The step size of EBSD observation was 0.2 μm, and the TSL OIM Analysis software (6.14, EDAX-TSL, Mahwah, NJ, USA) was used to analyze the data. Transmission electron microscopy (TEM, Tecnai G^2^F30, FEI, Hillsboro, OR, USA) operating at 300 kV was also used to identify the phases and deformation mechanism of the alloys. 

## 3. Results and Discussion

### 3.1. Hot Deformation Behavior of the Sintered Alloy

**Figure 2:**
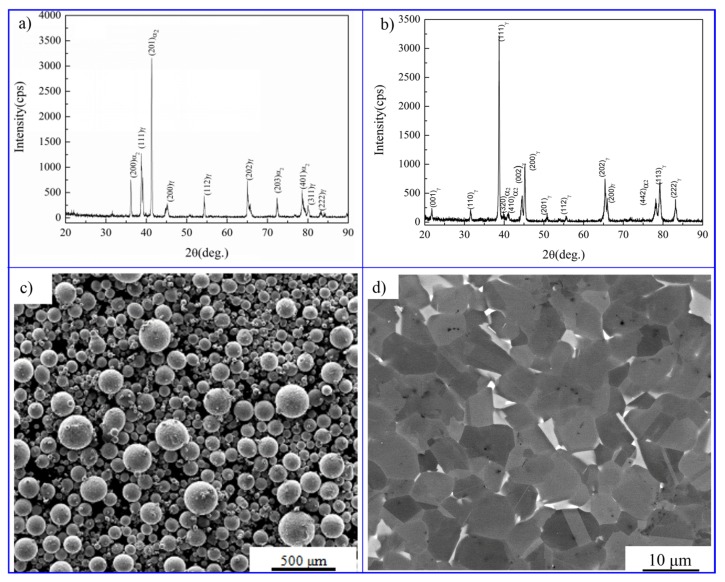
(**a**) X-ray diffraction (XRD) patterns of the pre-alloyed powders; (**b**) XRD patterns of the alloy sintered at 1300 °C; (**c**) morphology (scanning electron microscopy—SEM) of the pre-alloyed powders; (**d**) metallographic photograph (SEM) of the alloy sintered at 1300 °C.

Figure 2a shows that the pre-alloyed powders are composed of α_2_ and γ phase by X-ray diffraction (XRD) examination, and the diffraction peak of α_2_ phase is much stronger than γ phase, suggesting that there is a great proportion of α_2_ phase in the pre-alloyed powders. The microphotograph of the powders is displayed in Figure 2c, the particle size of the powders ranged from 5 to 300 μm. As shown in Figure 2b, after sintering, the alloy is composed of a great number of γ phase and a few of α_2_ phase, indicating the phase transition of α_2_→γ occurred during the sintering process. Figure 2d shows the metallographic photograph of the sintered alloy, it can be observed that an NG microstructure is obtained which is composed of plenty of equiaxed γ grains (grey phase in Figure 2d) and a few of α_2_ grains (white phase in Figure 2d). 

**Figure 3:**
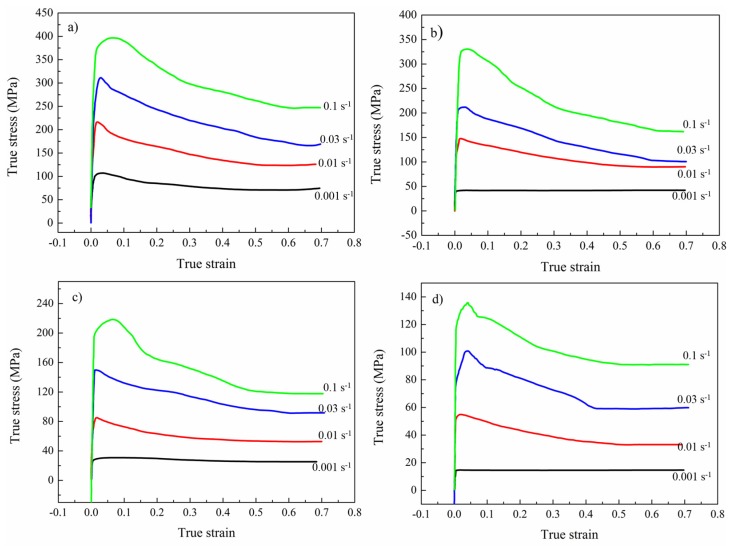
Compressive true stress-true strain curves of the TiAl based alloy (sintered at 1300 °C) tested at 1125 °C (**a**); 1175 °C (**b**); 1225 °C (**c**); and 1275 °C (**d**) with various strain rates.

The deformation behavior of γ-TiAl based alloy is extremely sensitive to temperature and strain rate. High strain rate, low temperature, and uneven temperature distribution lead to frequent cracking [27]. To obtain a crack-free pancake, thermal physical simulation experiments were conducted in the temperature range of 1125–1275 °C with the strain rate ranging from 0.001 s^−1^ to 0.1 s^−1^, and the height reduction of all specimens was 50%. Figure 3 exhibits the compression flow stress curves of sintered TiAl based alloys tested at different temperatures and strain rates. The true stress-true strain curves at different deformation parameters show a similar trend in this study. Due to work-hardening, the flow stress increases dramatically at the first deformation stage. Then, with increasing strain, due to the effect of working hardening and dynamic softening [28,29], the flow stress ascends gradually to the peak stress at a certain strain. Subsequently, because of the dynamic effects between the softening and work-hardening mechanism, a gradual steady stage of flow stress is achieved with the strain increasing. During the deformation process, work-hardening is compatible with dynamic softening during the hot deformation. In addition, with the increase of testing temperature and the decrease of strain rate, the flow stress dwindles [28,29].

Hot deformation is the process of thermal activation. In the process, the metal atoms are in severe thermal motion, which requires atomic energy to cross a “threshold”, named the thermal deformation activation energy (Q). The Zenner-Hollomon (Z) parameters were adopted to illustrate the dependence of flow stress on deformation strain rate and temperature, and their relationship can be expressed as follows [30]:

(1) ε˙exp(QRT)=A1σn1

(2) ε˙exp(QRT)=A2exp(βσ)

(3) ε˙exp(QRT)=A[sinh(ασ)]n

(4) $$\alpha =\beta /n_{1}$$

(5) Z=ε˙exp(QRT)

In the equations, *R* is the Boltzmann constant (8.314 J·(mol·K)^−1^), *T* is the deformation temperature (K), *Q* is the activation energy of deformation (J·mol^−1^), *σ* is the flow stress (MPa), $\dot{\varepsilon }$ is the strain rate (s^−1^). Furthermore, A, A_1_, A_2_, n, n_1_, α, and β are material constants. Equation (1) is the power law which could be applied under the conditions of a low stress (ασ < 0.8), while Equation (2) named the exponential law is applied in a high stress range (ασ > 0.8). Equation (3) which is titled the hyperbolic-sine law could be applied over a wide range of stress. To calculate the parameters, Equations (1)–(3) could be written as follows [28,29,30].

(6) lnε˙=n1lnσp+lnA1−QRT

(7) lnε˙=βσ+lnA2−QRT

(8) ln[sinh(ασp)]=−lnAn+lnε˙n+QnRT

**Figure 4:**
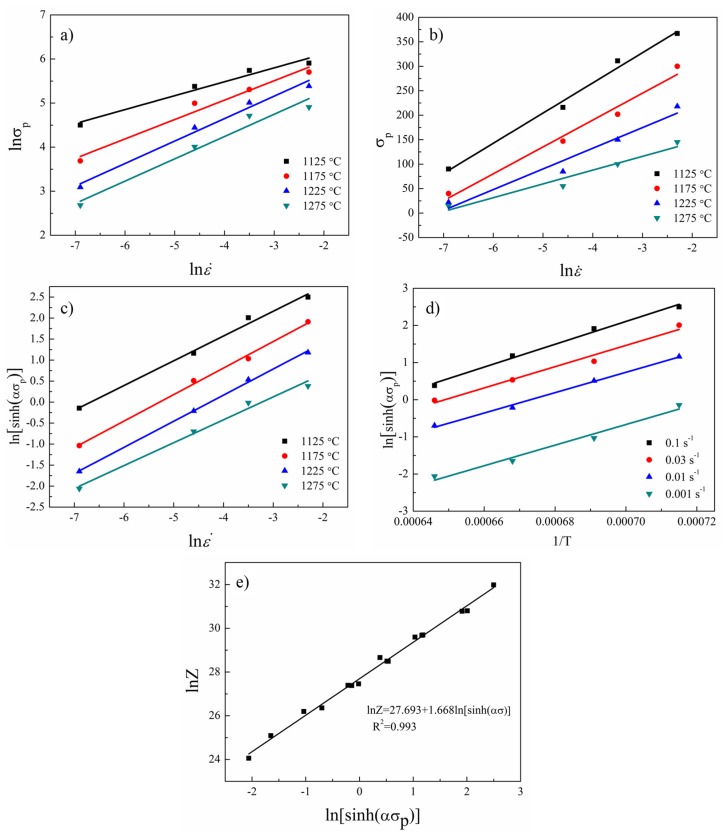
The linear relationship of ln$\dot{\varepsilon }$-ln*σ_p_* (**a**); ln$\dot{\varepsilon }$-*σ_p_* (**b**); ln$\dot{\varepsilon }$-ln[sinh(*ασ_p_*)] (**c**); 1/T-ln[sinh(*ασ_p_*)] (**d**); and lnZ-ln[sinh(*ασ_p_*)] (**e**).

Figure 4 shows the relationships of the ln$\dot{\varepsilon }$-ln*σ_p_*, ln$\dot{\varepsilon }$-*σ_p_*, ln$\dot{\varepsilon }$-ln[sinh(*ασ_p_*)], 1/T-ln[sinh(*ασ_p_*)], and lnZ-ln[sinh(*ασ_p_*)]. It was found that Equation (3) had a wider applicability in this research. The parameters which were calculated through the fitting lines are listed in Table 1, and the constitutive equation and *Z* parameter of the sintered TiAl based alloy can be expressed as:

(9) ε˙=1.064×1012exp(−398500RT)[sinh(8.7×10−3σ)]1.688

(10) Z=ε˙exp(QRT)=ε˙exp(398500RT)

### 3.2. The Processing Map and Microstructure Evolution

Processing map developed on the basis of the dynamic materials model (DMM) is a powerful way to study the deformation behaviors of TiAl alloys [17,18,19,20]. In the model, the total power of the work piece could be expressed as Equation (11):

(11) $$P=G+J=\int_{0}^{\dot{\varepsilon }}\sigma d\dot{\varepsilon }+\int_{0}^{\sigma }\dot{\varepsilon }d\sigma$$

In the equation, the power inputting the work piece during plastic processing is divided into two parts. *G* represents the power consumed in plastic deformation, while *J* is the power consumed in structural transformation. The $\dot{\varepsilon }$ and *σ* represent the strain rate and flow stress, respectively [17,18,19,20]. 

To characterize the microstructure evolution, the power dissipation efficiency (*η*) is used in the processing maps, which could be determined as Equation (11):

(12) $$\eta =\frac{2m}{m+1}$$

where *m* described as the strain rate sensitivity exponent could be calculated by Equation (12):

(13) $$m=\frac{\partial \mathrm{lg}\sigma }{\partial \mathrm{lg}\dot{\varepsilon }}|{}_{\varepsilon ,T}$$

A continuum criterion for the occurrence of flow instability proposed by Ziegler [29] using the principles of maximum rate of entropy production, is given by:

(14) $$\xi (\varepsilon )=\frac{\partial \ln(\frac{m}{m+1})}{\partial \ln\dot{\varepsilon }}+m<0$$

**Figure 5:**
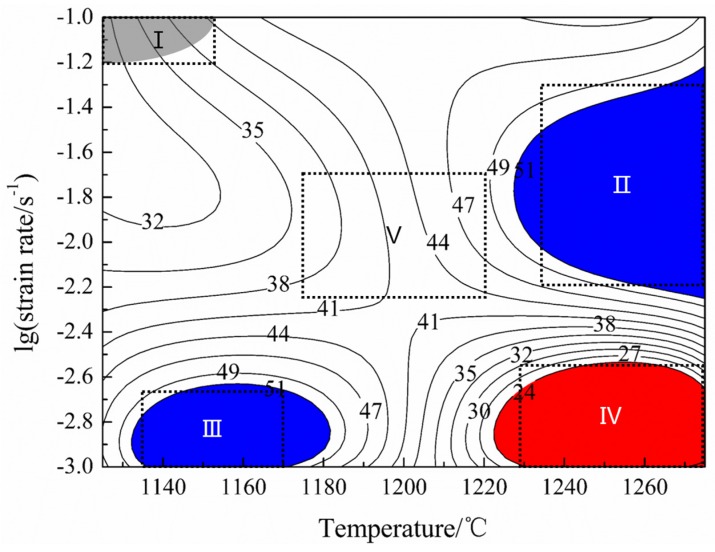
The processing map of sintered alloy with a true strain of 0.7.

Based on the DMM theory and the instability parameter ξ [17,18,19,20], the processing map for the isothermal compression of the sintered TiAl based alloy with the temperature ranging from 1125 to 1275 °C and the strain rate ranging from 0.001 to 0.1 s^−1^ with a true strain of 0.7, is shown in Figure 5. The contour lines represent the different η which could be described as the power dissipation efficiency in the processing map. In this study, the gray area indicates the instability region which was calculated by the instability parameter ξ. The red area with low η value is caused by the energy dissipation. The blue area indicates the high η regions, which might be the best hot deformation region. The narrow instability region of this alloy means an outstanding deformability in this study.

**Figure 6:**
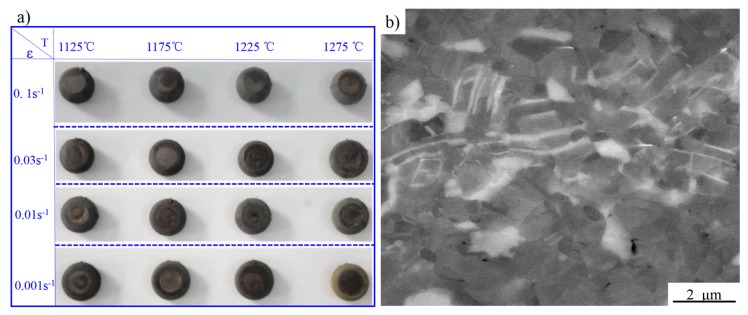
The macro morphology of the compressed specimens (**a**); and the micro-topography (SEM) of localized plastic flow occurring at 1125 °C/0.1 s^−1^ (**b**).

As shown in Figure 6a, no cracks could be observed in the compressive specimens. As displayed in Figure 6b, a localized plastic flow zone could be only observed in the 1125 °C (deformation temperature)/0.1 s^−1^ (strain rate) deformed specimen, indicating the sintered alloy obtained an excellent hot workability. Furthermore, the area of instability can be correctly identified by the parameter ξ.

**Figure 7:**
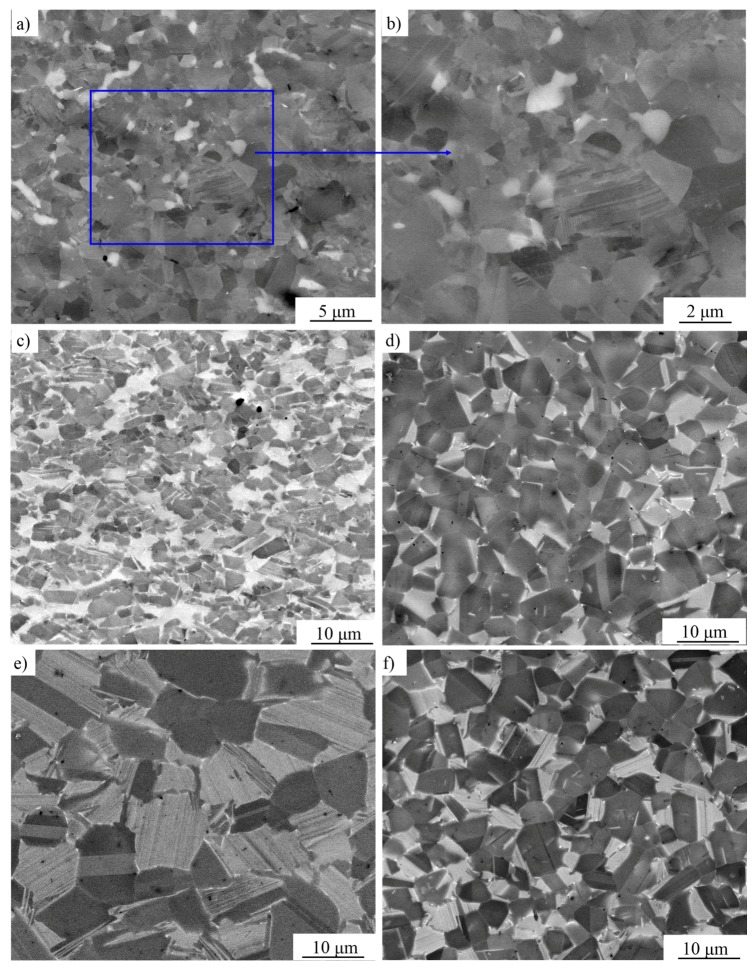
The microstructures (SEM) at the core of the specimens compressed under different parameters with a true strain of 0.7: (**a**,**b**) 1125 °C/0.1 s^−1^ (Figure 5I); (**c**) 1275 °C/0.03 s^−1^, (Figure 5II); (**d**) 1175 °C/0.001 s^−1^ (Figure 5III); (**e**) 1275 °C/0.001 s^−1^ (Figure 5IV); (**f**) 1200 °C/0.01 s^−1^ (Figure 5V).

In order to study the microstructure evolution and further optimize the deformation parameters, five specimens were observed by SEM. As shown in the processing map (Figure 5), the observed specimens were obtained at the low temperature–high strain rate area (I), the high temperature–middle strain rate area (II), the low temperature–low strain rate area (III), the high temperature–low strain rate area (IV), and the middle temperature–middle strain rate area (V), respectively. The microstructures obtained at different compression conditions are presented in Figure 7. After compression, it could be observed that all the microstructures of the specimens changed remarkably compared with the sintered alloy. The grain size, as well as the proportion of α_2_ phase changed with the variation of the testing temperature and strain rate. The microstructure tested in the low temperature–high strain rate area (I) is inhomogeneous, while the microstructure tested in the high temperature–low strain rate area (IV) is composed of equiaxial γ grains and α_2_/γ-colonies named by duplex microstructure. The specimens tested in the low temperature–low strain rate area (III) consist of refined equiaxial γ grains and α_2_ grains which means an extensive processing range. Although alloys compressed in the high temperature–middle strain rate area (II) present a high η, however, due to the high deformation temperature and relative high strain rate, the microstructure is composed of deformed γ grains and α_2_/γ-colonies with slight orientation, which would lead to relatively poor plasticity and anisotropy of the mechanical properties. The specimens tested in the middle temperature–middle strain rate area (V) are composed of γ grains and α_2_/γ-colonies. Considering the η and the microstructures of the compressed alloys, the best deformation range for the sintered TiAl alloy is in a temperature range of 1130–1180 °C and a strain rate range of 2.5 × 10^−3^–1 × 10^−3^ s^−1^.

### 3.3. The PCIF of the Sintered TiAl Based Alloy

**Figure 8:**
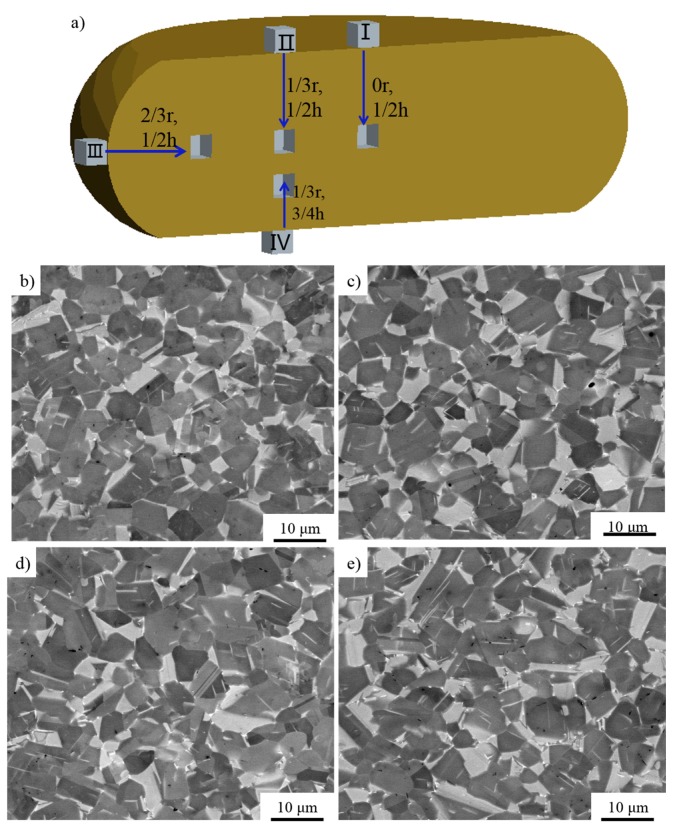
The sampling place (**a**), and the microstructures (SEM) of the forged alloy fabricated at 1175 °C with a nominal strain rate of 10^−3^ s^−1^ sampled at (**b**) I; (**c**) II; (**d**) III; and (**e**) IV.

Low temperature, high strain rate, and uneven temperature distribution would lead to cracking during the forging process [31] although traditional pack forging could reduce the temperature variation effectively by applying heat insulating materials and increasing the strain rate. Failure rate caused by cracking is still high due to the temperature variation and high strain rate. The isothermal forging process could provide a more uniform temperature distribution, although the alloys were forged under a low strain rate. However, there is limited equipment that can withstand a high temperature above 1100 °C. Hence, advanced forging technology should be developed which could be applied in the forging of TiAl based alloys. PCIF could reach this high temperature due to the fact that only the alloy is heated. In this research, forged TiAl alloy blocks without cracks were fabricated by using PCIF equipment (as shown in Figure 2) through one-step forging. The height reduction of the alloy was about 50%, the deformation temperature was about 1175 °C and the nominal strain rate was 10^−3^ s^−1^. During the forging process, in order to keep a constant temperature, the pulse current flowing through the specimen increased gradually with the specimen’s radius growth, and the forged disk was then cooled in a vacuum environment. After the forging process as shown schematically in Figure 8a, three different specimens were sectioned at a 0, 1/3, and 2/3 r horizontal distance from the center of the longitudinal section, respectively. In order to further study the uniformity of microstructure, another specimen was sectioned at a 2/3 r and 1/4 height of the forged alloy. It could be found that all the specimens were composed of refined NG microstructure (as shown in Figure 8b–e). In addition, compared with the sintered alloys, the volume of α_2_ phase increased seemingly.

**Figure 9:**
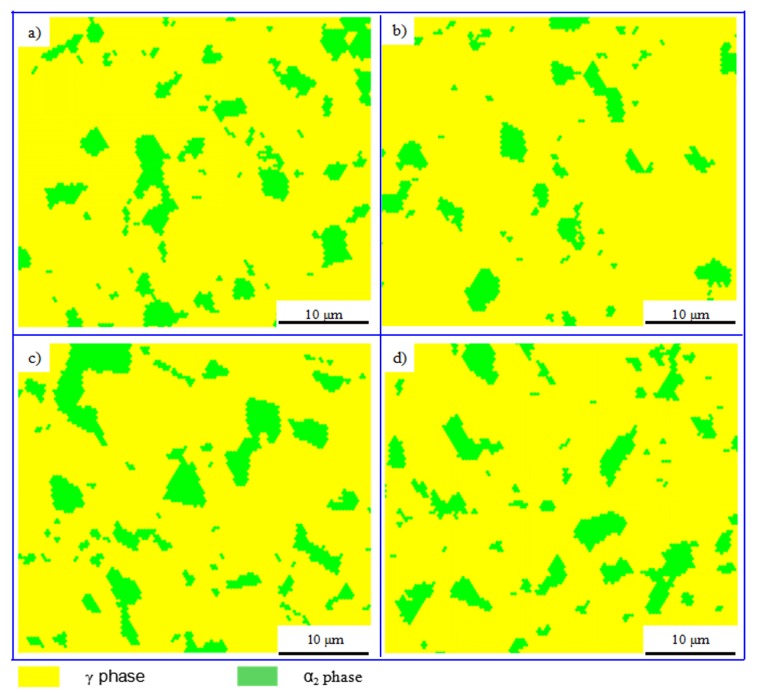
The phase distribution maps (electron backscatter diffraction—EBSD) of the specimens sampled at (**a**) I; (**b**) II; (**c**) III; and (**d**) IV as shown in Figure 8a.

The α_2_ phase could absorb more oxygen to improve the plastic property of γ-TiAl based alloy [32]. However, too much α_2_ phase will reduce the plastic property of TiAl based alloy because of the lacking sliding systems. When the volume proportion of α_2_ phase is about 10–15%, the γ-TiAl based alloy would obtain the best plastic property [32]. Figure 9 shows that the distribution of the α_2_ phase in the alloys (sampled as shown in Figure 8a) is homogenous. Furthermore, Table 2 shows that the volume proportion of α_2_ phase is in a range of 12.9–14.2%, while the α_2_ phase in sintered alloy is less than 5%, indicating that phase transformation of γ→α_2_ occurs during the PCIP process.

**Figure 10:**
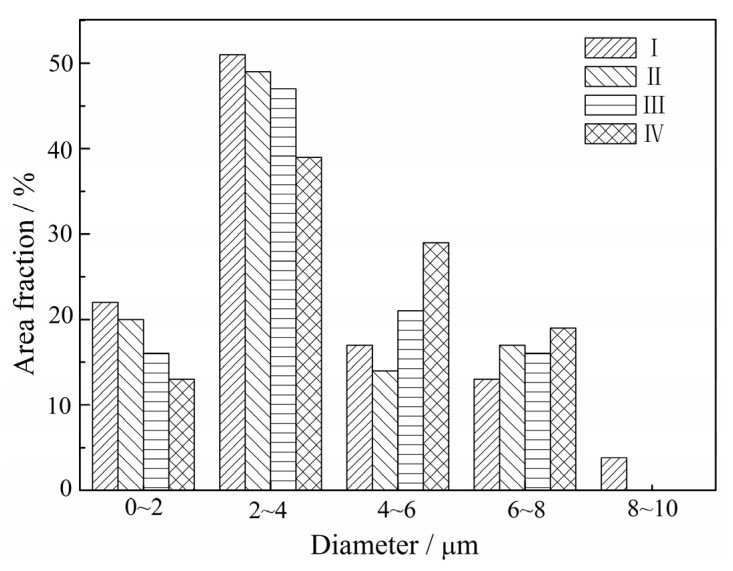
Grain size distribution of the specimens sampled at (**a**) I; (**b**) II; (**c**) III; and (**d**) IV as shown in Figure 8a.

Figure 10 displays the grain size distribution of the I–IV specimens (sampled as shown in Figure 8a). The grain size of the forged alloy sampled at different areas is nearly consistent, and all of their grain sizes are small and homogeneous compared with the sintered alloy. Additionally, most of them are in the range of 2–8 μm, suggesting that the forged alloy might possess good deformability under proper deformation conditions.

**Figure 11:**
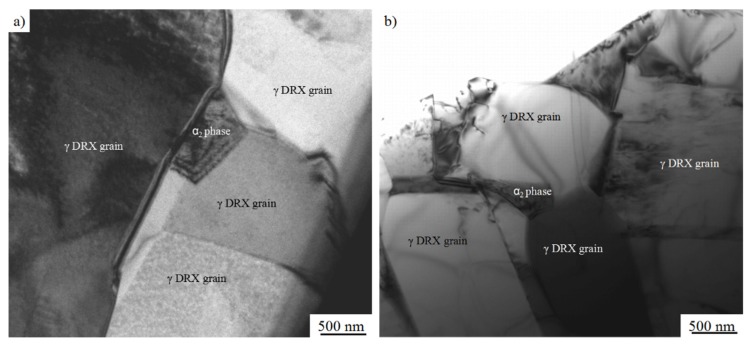
The transmission electron microscopy (TEM) images of specimens sampled at (**a**) I; (**b**) III as shown in Figure 8a.

In order to further confirm the microstructures and study the softening mechanism, specimens of I and III sampled from the forged alloy (as shown in Figure 8a) were observed by TEM. Figure 11 shows the TEM images of the forged alloys. Both of them consist of dynamic recrystallization (DRX) γ grains with few dislocations and a few α_2_ grains.

**Figure 12:**
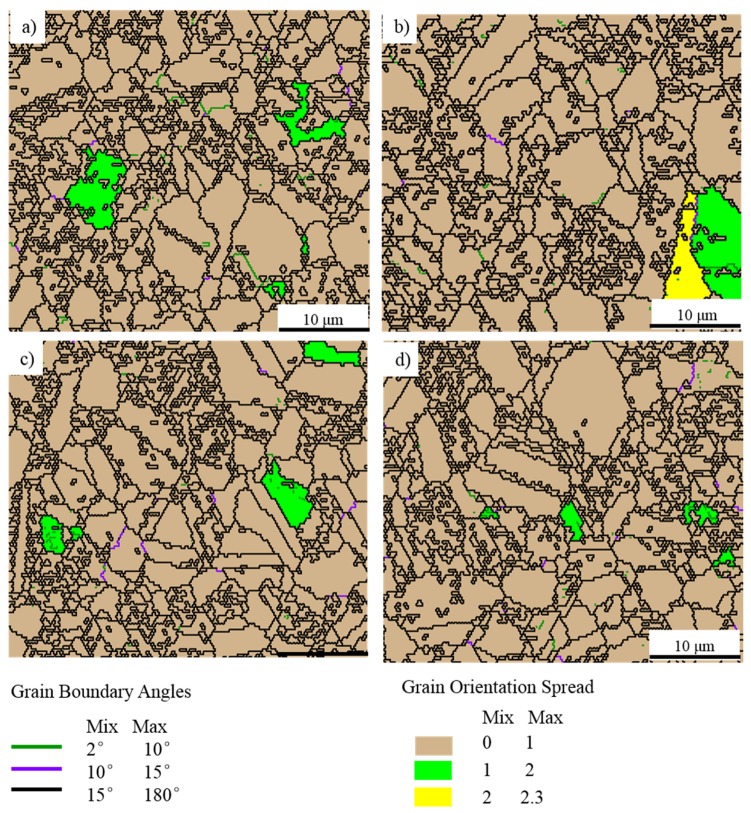
The grain boundaries and grain orientation spread (GOS) distribution maps (EBSD) of the specimens sampling at (**a**) I; (**b**) II; (**c**) III; and (**d**) IV as shown in Figure 8a.

The grain orientation spread (GOS) maps, which can be used in describing the average difference in orientation between the average grain orientation and all measurements in one grain [33,34,35], are shown in Figure 12. The GOS degree which is less than 2° could be used as an evaluation standard of DRX [33,34,35]. The volume of the grains of which the GOS degree is less than 2° is larger than 96%, and few low angle grain boundaries (LAGBs, 2°–10°) could be detected in the specimens. Furthermore, the TEM images (Figure 10) also shows few dislocations in the forged alloy. In conclusion, it can be inferred that complete DRX occurred during the PCIF process. The DRX mechanism could be divided into continuous dynamic recrystallization (CDRX) and discontinuous dynamic recrystallization (DDRX), and CDRX is generally generated through the rotation of subgrains. During the CDRX process, LAGBs transform into medium angle grain boundaries (MAGBs) and high angle grain boundaries (HAGBs) by absorbing dislocations, therefore, MAGBs could be used in identifying the CDRX and the DDRX mechanisms [33,34,35]. Figure 11 shows there are few MAGBs in the forged alloys, indicating that DDRX might be the main DRX mechanism.

**Figure 13:**
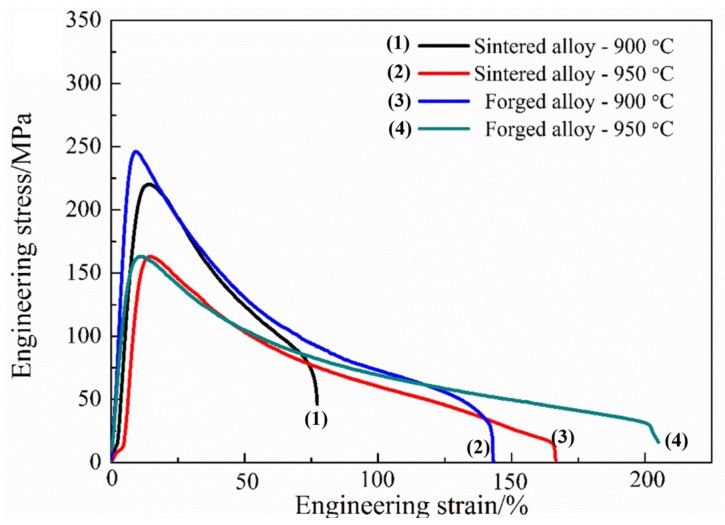
Tensile properties of the sintered and forged alloys tested at 900 °C and 950 °C with a strain rate of 2 × 10^−4^ s^−1^.

Uniform dynamic recrystallization microstructure, suitable proportion of α_2_ phase, and low dislocation density suggest that the forged alloy might obtain excellent hot deformability. Figure 13 displays the tensile properties of the sintered and forged alloys which were tested at a strain rate of 2 × 10^−4^ s^−1^ at 900 °C and 950 °C, respectively. The elongation (δ) of the forged alloy is 136% at 900 °C, while the sintered alloy’s δ is only 66% at 900 °C. The δ of forged alloy is 192% at 950 °C, meaning that it possesses good processing performance. The improved tensile properties of the forged alloy are mainly ascribed to two aspects: the improvement of density as well as the homogenization and refinement of microstructure caused by DRX.

## 4. Conclusions

In this research, the hot deformation behavior and the microstructure evolution of sintered TiAl based alloy were investigated. Accordingly, forged alloy was fabricated by PCIF processing at an appropriate deformation condition. The main conclusions were drawn as follows:

(1) Based on the compression tests, the Q of sintered TiAl based alloy was calculated to be 398.50 kJ/mol when compressed at a temperature range of 1125–1275 °C, and the constitutive equation was expressed as:
  ε˙=1.064×1012exp(−398500RT)[sinh(8.7×10−3σ)]1.688
(2) The sintered alloy possessed excellent deformation performance. The microstructural evolution depended strongly on the deformation temperature and strain rate. According to the processing map and microstructure observation, the optimal hot working parameters of the sintered alloy were in the temperature range of 1130–1180 °C and strain rate range of 10^−3^–2.5 × 10^−3^ s^−1^.
(3) The PCIF process produced forged TiAl based alloys with homogeneous and refined microstructure successfully. When the sintered alloy was forged at 1175 °C with a nominal strain rate of 10^−3^ s^−1^, phase transformation of γ→α_2_ and complete DRX occurred, resulting in obtaining a refined NG microstructure which possessed a good secondary hot workability. At 900 °C, the elongation of the forged alloy was double that of the as-sintered one.

## Figures and Tables

**Table 1:** Constitutive equation parameters of the alloy.

n_1_	β	Q	α	A	n
1.976	0.0172	398,500	8.7 × 10^−3^	1.064 × 10^12^	1.688

**Table 2:** The γ and α_2_ phase fractions of the specimens sampled at (a) I;, (b) II, (c) III, and (d) IV as shown in the Figure 10a.

Sample	I	II	III	IV
γ phase	86.1	87.1	85.8	86.4
α_2_ phase	13.9	12.9	14.2	13.6
